# *catena*-Poly[[tetra­aqua­copper(II)]-μ-pyrazine-2-carboxamide-κ^3^ *N* ^4^:*N* ^1^,*O*-[bis­(sulfato-κ*O*)copper(II)]-μ-pyrazine-2-carboxamide-κ^3^ *N* ^1^,*O*:*N* ^4^]

**DOI:** 10.1107/S1600536812031844

**Published:** 2012-07-18

**Authors:** Sadif A. Shirvan, Sara Haydari Dezfuli

**Affiliations:** a Department of Chemistry, Omidieh Branch, Islamic Azad University, Omidieh, Iran

## Abstract

In the crystal of the title polymeric compound, [Cu_2_(SO_4_)_2_(C_5_H_5_N_3_O)_2_(H_2_O)_4_]_*n*_, two independent Cu^II^ atoms are located on individual inversion centers. One Cu^II^ atom is coordinated by four water mol­ecules and two pyrazine-2-carboxamide ligands in a distorted O_4_N_2_ octa­hedral geometry; the other is *N*,*O*-chelated by two pyrazine-2-carboxamide ligands and further coordinated by two sulfate anions in a distorted O_4_N_2_ octa­hedral geometry. The pyrazine-2-carboxamide ligands bridge the Cu^II^ atoms to form a polymeric chain running along [110]. The crystal structure features N—H⋯O, O—H⋯O and weak C—H⋯O hydrogen bonds.

## Related literature

For related structures, see: Abu-Youssef *et al.* (2006 ▶); Azhdari Tehrani *et al.* (2010 ▶); Goher & Mautner (2000 ▶); Kristiansson (2002 ▶); Mir Mohammad Sadegh *et al.* (2010 ▶); Munakata *et al.* (1997 ▶); Pacigova *et al.* (2008 ▶); Shirvan & Haydari Dezfuli (2012*a* ▶,*b* ▶,*c* ▶).
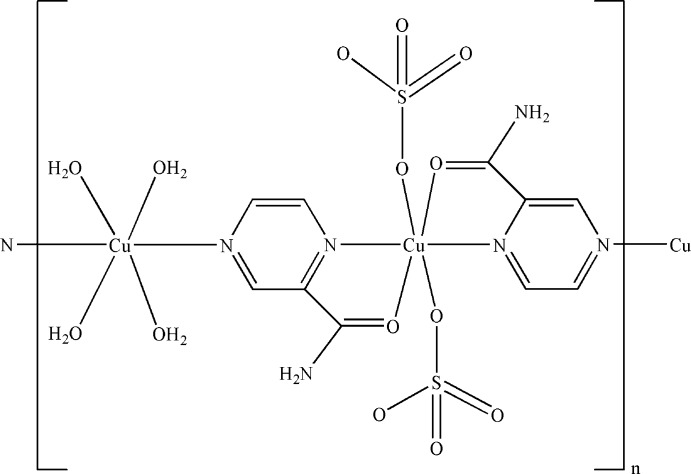


## Experimental

### 

#### Crystal data

[Cu_2_(SO_4_)_2_(C_5_H_5_N_3_O)_2_(H_2_O)_4_]
*M* *_r_* = 318.77
Monoclinic, 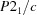

*a* = 11.2699 (12) Å
*b* = 7.3799 (7) Å
*c* = 11.8669 (15) Å
β = 95.267 (9)°
*V* = 982.81 (19) Å^3^
*Z* = 4
Mo *K*α radiation
μ = 2.47 mm^−1^
*T* = 298 K
0.25 × 0.20 × 0.04 mm

#### Data collection

Bruker APEXII CCD area-detector diffractometer
Absorption correction: multi-scan (*SADABS*; Bruker, 2001 ▶) *T*_min_ = 0.589, *T*_max_ = 0.926
7420 measured reflections
1928 independent reflections
1544 reflections with *I* > 2σ(*I*)
*R*_int_ = 0.090

#### Refinement

*R*[*F* ^2^ > 2σ(*F* ^2^)] = 0.035
*wR*(*F* ^2^) = 0.103
*S* = 1.07
1928 reflections
173 parameters
6 restraints
H atoms treated by a mixture of independent and constrained refinement
Δρ_max_ = 0.83 e Å^−3^
Δρ_min_ = −0.69 e Å^−3^

### 

Data collection: *APEX2* (Bruker, 2007 ▶); cell refinement: *SAINT* (Bruker, 2007 ▶); data reduction: *SAINT*; program(s) used to solve structure: *SHELXS97* (Sheldrick, 2008 ▶); program(s) used to refine structure: *SHELXL97* (Sheldrick, 2008 ▶); molecular graphics: *SHELXTL* (Sheldrick, 2008 ▶); software used to prepare material for publication: *SHELXTL*.

## Supplementary Material

Crystal structure: contains datablock(s) I, global. DOI: 10.1107/S1600536812031844/xu5585sup1.cif

Structure factors: contains datablock(s) I. DOI: 10.1107/S1600536812031844/xu5585Isup2.hkl

Additional supplementary materials:  crystallographic information; 3D view; checkCIF report

## Figures and Tables

**Table 1:** Selected bond lengths (Å)

Cu1—O1	1.961 (3)
Cu1—O6	2.447 (3)
Cu1—N1	1.979 (3)
Cu2—O2	2.363 (2)
Cu2—O3	1.968 (3)
Cu2—N2	2.034 (2)

**Table 2:** Hydrogen-bond geometry (Å, °)

*D*—H⋯*A*	*D*—H	H⋯*A*	*D*⋯*A*	*D*—H⋯*A*
O2—H2*B*⋯O4^i^	0.83 (3)	2.01 (4)	2.845 (4)	177 (5)
O2—H2*C*⋯O7^ii^	0.83 (4)	2.03 (3)	2.815 (4)	159 (3)
O3—H3*D*⋯O7^iii^	0.82 (3)	1.87 (3)	2.687 (3)	175 (5)
O3—H3*E*⋯O4^ii^	0.82 (3)	1.87 (3)	2.679 (4)	169 (3)
N3—H3*B*⋯O6^iv^	0.86	2.01	2.820 (4)	157
N3—H3*C*⋯O5^v^	0.86	2.03	2.862 (5)	162
C1—H1⋯O7^vi^	0.93	2.24	3.108 (5)	154

Symmetry codes: (i) 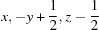; (ii) 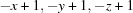; (iii) 
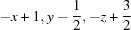
; (iv) 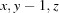; (v) 
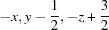
; (vi) 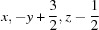.

**Fig. 1.**
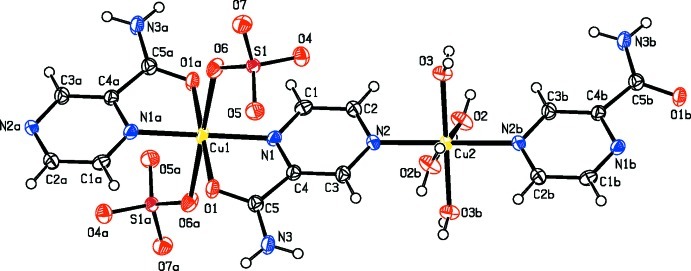
The molecular structure of the title molecule, with the atom-numbering scheme. Displacement ellipsoids are drawn at the 30% probability level. [Symmetry codes: (a) -x,1 - y,1 - z; (b) 1 - x,-y,1 - z].

**Fig. 2.**
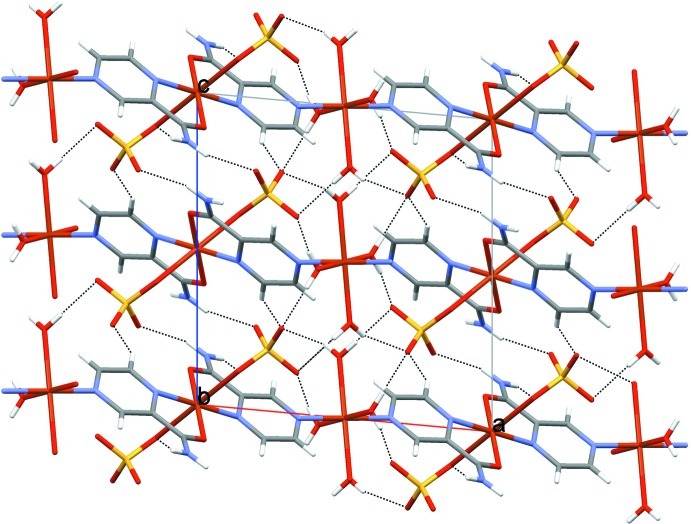
Unit-cell packing diagram for title molecule. Hydrogen bonds are shown as dashed lines.

## supplementary crystallographic information

### Comment

Pyrazine-2-carboxamide (pzc) is a good ligand, and a few complexes with pzc have been prepared, such as that of mercury (Azhdari Tehrani *et al.*, 2010; Mir Mohammad Sadegh *et al.*, 2010), vanadium (Pacigova *et al.*, 2008), manganese (Abu-Youssef *et al.*, 2006), copper (Kristiansson, 2002; Munakata *et al.*, 1997; Goher & Mautner, 2000), zinc (Shirvan & Haydari Dezfuli, 2012*a*,*b*,*c*). Here, we report the synthesis and structure of the title compound.

The asymmetric unit of the title compound, (Fig. 1), contains two half of Cu^II^ atom, two half of pyrazine-2-carboxamide ligands, two water molecules and one sulfate anion. The octahedral Cu^II^ ions form a polymeric chain, being bridged by two pyrazine-2-carboxamide ligands. There are two crystallographically independent Cu^II^ centers with center adopts a {CuO_4_N_2_} coordination geometry, both of which reside on centers of symmetry. The first Cu(1) center defined by two oxygen and two nitrogen from two pyrazine-2-carboxamide ligands and by two oxygen from two sulfate anions. The second Cu(2) center is also found in a octahedral coordination environment by two pyrazine nitrogen donors from two pyrazine-2-carboxamide ligands and by four aquo oxygen donors. The Cu—O and Cu—N bond lengths and angles are collected in Table 1.

In the crystal structure, Intermolecular N—H···O, O—H···O and C—H···O hydrogen bonds may stabilize the structure (Table 2 & Fig. 2).

### Experimental

A solution of pyrazine-2-carboxamide (0.25 g, 2.0 mmol) in methanol (10 ml) was added to a solution of CuSO_4_.5H_2_O (0.25 g, 1.0 mmol) in water (5 ml) and the resulting blue solution was stirred for 15 min at room temperature. This solution was left to evaporate slowly at room temperature. After one week, blue block crystals of the title compound were isolated (yield 0.23 g, 72.2%).

### Refinement

Water H atoms were located on a difference Fourier map and refined isotroipcally. Other H atoms were positioned geometrically with C—H = 0.93 and N—H = 0.86 Å, and constrained to ride on their parent atoms with *U*_iso_(H) = 1.2*U*_eq_(C,N).

### Crystal data

**Table:**

[Cu_2_(SO_4_)_2_(C_5_H_5_N_3_O)_2_(H_2_O)_4_]	*F*(000) = 644
*M**_r_* = 318.77	*D*_x_ = 2.154 Mg m^−^^3^
Monoclinic, *P*2_1_/*c*	Mo *K*α radiation, λ = 0.71073 Å
Hall symbol: -P 2ybc	Cell parameters from 7420 reflections
*a* = 11.2699 (12) Å	θ = 1.8–26.0°
*b* = 7.3799 (7) Å	µ = 2.47 mm^−^^1^
*c* = 11.8669 (15) Å	*T* = 298 K
β = 95.267 (9)°	Plate, blue
*V* = 982.81 (19) Å^3^	0.25 × 0.20 × 0.04 mm
*Z* = 4	

### Data collection

**Table:**

Bruker APEXII CCD area-detector diffractometer	1928 independent reflections
Radiation source: fine-focus sealed tube	1544 reflections with *I* > 2σ(*I*)
Graphite monochromator	*R*_int_ = 0.090
ω scans	θ_max_ = 26.0°, θ_min_ = 1.8°
Absorption correction: multi-scan (*SADABS*; Bruker, 2001)	*h* = −13→13
*T*_min_ = 0.589, *T*_max_ = 0.926	*k* = −8→9
7420 measured reflections	*l* = −14→14

### Refinement

**Table:**

Refinement on *F*^2^	Primary atom site location: structure-invariant direct methods
Least-squares matrix: full	Secondary atom site location: difference Fourier map
*R*[*F*^2^ > 2σ(*F*^2^)] = 0.035	Hydrogen site location: inferred from neighbouring sites
*wR*(*F*^2^) = 0.103	H atoms treated by a mixture of independent and constrained refinement
*S* = 1.07	*w* = 1/[σ^2^(*F*_o_^2^) + (0.057*P*)^2^] where *P* = (*F*_o_^2^ + 2*F*_c_^2^)/3
1928 reflections	(Δ/σ)_max_ = 0.006
173 parameters	Δρ_max_ = 0.83 e Å^−^^3^
6 restraints	Δρ_min_ = −0.69 e Å^−^^3^

### Special details

**Table:**

Geometry. All e.s.d.'s (except the e.s.d. in the dihedral angle between two l.s. planes) are estimated using the full covariance matrix. The cell e.s.d.'s are taken into account individually in the estimation of e.s.d.'s in distances, angles and torsion angles; correlations between e.s.d.'s in cell parameters are only used when they are defined by crystal symmetry. An approximate (isotropic) treatment of cell e.s.d.'s is used for estimating e.s.d.'s involving l.s. planes.
Refinement. Refinement of *F*^2^ against ALL reflections. The weighted *R*-factor *wR* and goodness of fit *S* are based on *F*^2^, conventional *R*-factors *R* are based on *F*, with *F* set to zero for negative *F*^2^. The threshold expression of *F*^2^ > σ(*F*^2^) is used only for calculating *R*-factors(gt) *etc*. and is not relevant to the choice of reflections for refinement. *R*-factors based on *F*^2^ are statistically about twice as large as those based on *F*, and *R*- factors based on ALL data will be even larger.

### Fractional atomic coordinates and isotropic or equivalent isotropic displacement parameters (Å^2^)

**Table:**

	*x*	*y*	*z*	*U*_iso_*/*U*_eq_	
C1	0.2270 (3)	0.3902 (5)	0.4101 (3)	0.0263 (7)	
H1	0.2189	0.4879	0.3605	0.032*	
C2	0.3319 (3)	0.2877 (5)	0.4182 (3)	0.0272 (8)	
H2	0.3936	0.3209	0.3756	0.033*	
C3	0.2541 (3)	0.1005 (5)	0.5473 (3)	0.0262 (8)	
H3	0.2609	0.0001	0.5948	0.031*	
C4	0.1517 (3)	0.2024 (5)	0.5411 (3)	0.0230 (7)	
C5	0.0473 (3)	0.1773 (5)	0.6079 (3)	0.0261 (7)	
N1	0.1397 (2)	0.3496 (4)	0.4721 (2)	0.0230 (6)	
N2	0.3451 (2)	0.1431 (4)	0.4860 (2)	0.0229 (6)	
N3	0.0314 (3)	0.0226 (4)	0.6589 (3)	0.0333 (8)	
H3C	−0.0296	0.0077	0.6965	0.040*	
H3B	0.0820	−0.0639	0.6547	0.040*	
O1	−0.0233 (2)	0.3090 (4)	0.6107 (2)	0.0281 (6)	
O2	0.5178 (3)	0.0396 (4)	0.3047 (2)	0.0364 (7)	
H2B	0.465 (3)	0.007 (6)	0.255 (3)	0.040 (13)*	
H2C	0.562 (3)	0.107 (5)	0.272 (3)	0.045 (14)*	
O3	0.5887 (2)	0.2275 (4)	0.5292 (2)	0.0280 (6)	
H3D	0.627 (4)	0.248 (7)	0.5901 (17)	0.069 (18)*	
H3E	0.620 (3)	0.286 (5)	0.481 (2)	0.033 (12)*	
O4	0.3372 (2)	0.5564 (4)	0.6316 (2)	0.0313 (6)	
O5	0.2000 (3)	0.4720 (4)	0.7649 (2)	0.0364 (7)	
O6	0.1397 (2)	0.6869 (4)	0.6196 (2)	0.0330 (6)	
O7	0.2862 (2)	0.7724 (4)	0.7689 (2)	0.0340 (6)	
Cu1	0.0000	0.5000	0.5000	0.0258 (2)	
Cu2	0.5000	0.0000	0.5000	0.02044 (18)	
S1	0.23960 (7)	0.61972 (11)	0.69724 (6)	0.0199 (2)	

### Atomic displacement parameters (Å^2^)

**Table:**

	*U*^11^	*U*^22^	*U*^33^	*U*^12^	*U*^13^	*U*^23^
C1	0.0328 (19)	0.0213 (18)	0.0248 (16)	−0.0008 (15)	0.0027 (14)	0.0046 (14)
C2	0.0224 (17)	0.028 (2)	0.0322 (18)	0.0006 (15)	0.0065 (14)	0.0026 (15)
C3	0.0231 (17)	0.026 (2)	0.0297 (16)	0.0046 (15)	0.0026 (13)	0.0052 (15)
C4	0.0208 (17)	0.0205 (18)	0.0272 (16)	0.0014 (13)	−0.0011 (13)	−0.0019 (14)
C5	0.0228 (17)	0.0268 (19)	0.0282 (16)	0.0018 (14)	0.0000 (14)	−0.0001 (15)
N1	0.0220 (14)	0.0189 (15)	0.0275 (14)	0.0025 (11)	−0.0019 (12)	0.0008 (11)
N2	0.0162 (13)	0.0230 (16)	0.0294 (14)	0.0010 (11)	0.0021 (11)	0.0023 (12)
N3	0.0307 (17)	0.0274 (18)	0.0445 (18)	0.0118 (13)	0.0175 (15)	0.0125 (15)
O1	0.0254 (12)	0.0270 (14)	0.0325 (12)	0.0096 (10)	0.0050 (10)	0.0026 (11)
O2	0.0283 (14)	0.0500 (19)	0.0305 (13)	−0.0121 (13)	0.0003 (12)	0.0030 (13)
O3	0.0292 (14)	0.0275 (15)	0.0263 (12)	−0.0081 (11)	−0.0023 (11)	0.0035 (11)
O4	0.0297 (13)	0.0359 (15)	0.0290 (12)	0.0042 (11)	0.0068 (11)	0.0007 (11)
O5	0.0350 (15)	0.0319 (16)	0.0429 (15)	0.0014 (12)	0.0080 (12)	0.0126 (12)
O6	0.0321 (14)	0.0281 (15)	0.0358 (13)	0.0082 (11)	−0.0135 (11)	−0.0032 (11)
O7	0.0360 (14)	0.0310 (16)	0.0336 (13)	−0.0032 (12)	−0.0044 (11)	−0.0098 (12)
Cu1	0.0225 (3)	0.0205 (4)	0.0344 (3)	0.0111 (2)	0.0028 (2)	0.0033 (2)
Cu2	0.0126 (3)	0.0198 (3)	0.0290 (3)	0.0020 (2)	0.0018 (2)	0.0008 (2)
S1	0.0176 (4)	0.0204 (4)	0.0212 (4)	0.0009 (3)	−0.0011 (3)	0.0001 (3)

### Geometric parameters (Å, º)

**Table:**

Cu1—O1	1.961 (3)	O2—H2B	0.83 (3)
Cu1—O6	2.447 (3)	O2—H2C	0.83 (4)
Cu1—N1	1.979 (3)	O3—H3D	0.82 (3)
Cu1—O1^i^	1.961 (3)	O3—H3E	0.82 (3)
Cu1—O6^i^	2.447 (3)	N1—C1	1.316 (4)
Cu1—N1^i^	1.979 (3)	N1—C4	1.360 (5)
Cu2—O2	2.363 (2)	N2—C2	1.337 (5)
Cu2—O3	1.968 (3)	N2—C3	1.348 (4)
Cu2—N2	2.034 (2)	N3—C5	1.312 (5)
Cu2—O2^ii^	2.363 (2)	N3—H3C	0.8600
Cu2—O3^ii^	1.968 (3)	N3—H3B	0.8600
Cu2—N2^ii^	2.034 (2)	C1—C2	1.399 (5)
S1—O4	1.481 (2)	C3—C4	1.374 (5)
S1—O5	1.449 (3)	C4—C5	1.489 (5)
S1—O6	1.474 (3)	C1—H1	0.9300
S1—O7	1.479 (3)	C2—H2	0.9300
O1—C5	1.258 (4)	C3—H3	0.9300
			
S1···C4	3.683 (4)	O7···H2C^vi^	2.03 (3)
S1···O2^iii^	3.479 (3)	O7···H3D^vii^	1.87 (3)
S1···H3B^iv^	2.9500	O7···H1^x^	2.2400
S1···H3C^v^	2.9000	O7···H2B^iii^	2.90 (4)
S1···H3^iv^	3.0800	N1···S1	3.439 (3)
S1···H2C^vi^	3.01 (4)	N1···O1	2.593 (3)
S1···H3E^vi^	2.84 (3)	N1···O4	3.177 (4)
S1···H3D^vii^	2.97 (3)	N1···O6	3.043 (4)
S1···H2B^iii^	2.74 (4)	N1···N2	2.764 (4)
O1···O5	3.207 (4)	N1···C5	2.368 (4)
O1···N1	2.593 (3)	N1···O1^i^	2.966 (4)
O1···C4	2.343 (4)	N1···O6^i^	3.247 (3)
O1···N3^v^	3.165 (4)	N2···O2	3.126 (4)
O1···C1^i^	3.187 (4)	N2···O3	2.816 (3)
O1···O6^i^	2.921 (3)	N2···N1	2.764 (4)
O1···N1^i^	2.966 (4)	N2···O2^ii^	3.110 (4)
O2···O4^viii^	2.845 (4)	N2···O3^ii^	2.845 (4)
O2···N2^ii^	3.110 (4)	N2···O5^viii^	3.081 (4)
O2···O3	3.044 (4)	N3···O6^xi^	2.820 (4)
O2···N2	3.126 (4)	N3···C4^xii^	3.428 (5)
O2···C2	3.174 (5)	N3···O1^xiii^	3.165 (4)
O2···S1^viii^	3.479 (3)	N3···O5^xiii^	2.862 (5)
O2···O3^ii^	3.106 (4)	N3···H3	2.7700
O2···O7^vi^	2.815 (4)	C1···O4	3.059 (4)
O2···C3^ii^	3.152 (5)	C1···O5^viii^	3.180 (5)
O3···N2^ii^	2.845 (4)	C1···O7^xiv^	3.108 (5)
O3···O7^ix^	2.687 (3)	C2···O4	3.213 (4)
O3···C2	3.100 (4)	C2···O5^viii^	2.949 (5)
O3···O4^vi^	2.679 (4)	C3···O5^viii^	3.393 (4)
O3···O2	3.044 (4)	C4···O5	3.323 (4)
O3···N2	2.816 (3)	C4···S1	3.683 (4)
O3···O2^ii^	3.106 (4)	C4···N3^xii^	3.428 (5)
O3···C3^ii^	3.181 (4)	C5···O5	3.252 (5)
O4···N1	3.177 (4)	C3···H3B	2.7000
O4···C2	3.213 (4)	H1···O1^i^	2.7100
O4···O2^iii^	2.845 (4)	H1···O7^xiv^	2.2400
O4···O3^vi^	2.679 (4)	H2···O2	2.6800
O4···C1	3.059 (4)	H2···O3	2.8100
O5···C2^iii^	2.949 (5)	H2B···S1^viii^	2.74 (4)
O5···C1^iii^	3.180 (5)	H2B···O4^viii^	2.01 (4)
O5···C5	3.252 (5)	H2B···O7^viii^	2.90 (4)
O5···N2^iii^	3.081 (4)	H2C···S1^vi^	3.01 (4)
O5···C4	3.323 (4)	H2C···O7^vi^	2.03 (3)
O5···O1	3.207 (4)	H2C···H3^ii^	2.5500
O5···C3^iii^	3.393 (4)	H2C···H3D^viii^	2.58 (5)
O5···N3^v^	2.862 (5)	H3···S1^xi^	3.0800
O6···N1^i^	3.247 (3)	H3···O6^xi^	2.7100
O6···N3^iv^	2.820 (4)	H3···O7^xi^	2.6600
O6···N1	3.043 (4)	H3···N3	2.7700
O6···O1^i^	2.921 (3)	H3···H3B	2.2500
O6···C5^i^	3.419 (4)	H3···O2^ii^	2.6800
O7···O3^vii^	2.687 (3)	H3···O3^ii^	2.8800
O7···C1^x^	3.108 (5)	H3···H2C^ii^	2.5500
O7···O2^vi^	2.815 (4)	H3B···S1^xi^	2.9500
O1···H3C^v^	2.7400	H3B···O6^xi^	2.0100
O1···H1^i^	2.7100	H3B···O7^xi^	2.8300
O2···H3^ii^	2.6800	H3B···C3	2.7000
O2···H2	2.6800	H3B···H3	2.2500
O3···H2	2.8100	H3C···S1^xiii^	2.9000
O3···H3^ii^	2.8800	H3C···O1^xiii^	2.7400
O4···H2B^iii^	2.01 (4)	H3C···O5^xiii^	2.0300
O4···H3E^vi^	1.87 (3)	H3D···S1^ix^	2.97 (3)
O5···H3C^v^	2.0300	H3D···O7^ix^	1.87 (3)
O6···H3B^iv^	2.0100	H3D···H2C^iii^	2.58 (5)
O6···H3^iv^	2.7100	H3E···S1^vi^	2.84 (3)
O7···H3^iv^	2.6600	H3E···O4^vi^	1.87 (3)
O7···H3B^iv^	2.8300		
			
O1—Cu1—O6	97.77 (10)	O5—S1—O7	111.47 (15)
O1—Cu1—N1	82.32 (10)	O6—S1—O7	108.29 (16)
O1—Cu1—O1^i^	180.00	Cu1—O1—C5	114.4 (2)
O1—Cu1—O6^i^	82.23 (10)	Cu1—O6—S1	125.95 (17)
O1—Cu1—N1^i^	97.68 (10)	Cu2—O2—H2B	123 (3)
O6—Cu1—N1	86.20 (10)	Cu2—O2—H2C	130 (3)
O1^i^—Cu1—O6	82.23 (10)	H2B—O2—H2C	105 (4)
O6—Cu1—O6^i^	180.00	Cu2—O3—H3D	122 (3)
O6—Cu1—N1^i^	93.80 (10)	Cu2—O3—H3E	125 (2)
O1^i^—Cu1—N1	97.68 (10)	H3D—O3—H3E	107 (4)
O6^i^—Cu1—N1	93.80 (10)	Cu1—N1—C4	112.6 (2)
N1—Cu1—N1^i^	180.00	C1—N1—C4	118.7 (3)
O1^i^—Cu1—O6^i^	97.77 (10)	Cu1—N1—C1	127.8 (2)
O1^i^—Cu1—N1^i^	82.32 (10)	C2—N2—C3	117.6 (3)
O6^i^—Cu1—N1^i^	86.20 (10)	Cu2—N2—C2	120.7 (2)
O2—Cu2—O3	88.84 (10)	Cu2—N2—C3	121.7 (2)
O2—Cu2—N2	90.30 (10)	C5—N3—H3C	120.00
O2—Cu2—O2^ii^	180.00	C5—N3—H3B	120.00
O2—Cu2—O3^ii^	91.16 (10)	H3B—N3—H3C	120.00
O2—Cu2—N2^ii^	89.70 (10)	N1—C1—C2	120.6 (3)
O3—Cu2—N2	89.41 (11)	N2—C2—C1	121.3 (3)
O2^ii^—Cu2—O3	91.16 (10)	N2—C3—C4	121.4 (3)
O3—Cu2—O3^ii^	180.00	N1—C4—C5	112.3 (3)
O3—Cu2—N2^ii^	90.59 (11)	C3—C4—C5	127.2 (3)
O2^ii^—Cu2—N2	89.70 (10)	N1—C4—C3	120.4 (3)
O3^ii^—Cu2—N2	90.59 (11)	N3—C5—C4	120.1 (3)
N2—Cu2—N2^ii^	180.00	O1—C5—N3	123.1 (3)
O2^ii^—Cu2—O3^ii^	88.84 (10)	O1—C5—C4	116.8 (3)
O2^ii^—Cu2—N2^ii^	90.30 (10)	N1—C1—H1	120.00
O3^ii^—Cu2—N2^ii^	89.41 (11)	C2—C1—H1	120.00
O4—S1—O5	109.61 (17)	N2—C2—H2	119.00
O4—S1—O6	109.82 (14)	C1—C2—H2	119.00
O4—S1—O7	107.53 (14)	N2—C3—H3	119.00
O5—S1—O6	110.07 (17)	C4—C3—H3	119.00
			
O6—Cu1—O1—C5	−91.3 (2)	O3^ii^—Cu2—N2—C3	53.3 (3)
N1—Cu1—O1—C5	−6.2 (2)	O5—S1—O6—Cu1	−47.0 (2)
O6^i^—Cu1—O1—C5	88.7 (2)	O7—S1—O6—Cu1	−169.06 (14)
N1^i^—Cu1—O1—C5	173.8 (2)	O4—S1—O6—Cu1	73.8 (2)
O1—Cu1—O6—S1	41.74 (18)	Cu1—O1—C5—C4	12.7 (4)
N1—Cu1—O6—S1	−39.97 (17)	Cu1—O1—C5—N3	−166.3 (3)
O1^i^—Cu1—O6—S1	−138.26 (18)	C4—N1—C1—C2	−2.3 (5)
N1^i^—Cu1—O6—S1	140.03 (17)	Cu1—N1—C1—C2	166.0 (3)
O1—Cu1—N1—C4	−1.9 (2)	Cu1—N1—C4—C5	8.4 (4)
O6—Cu1—N1—C4	96.4 (2)	C1—N1—C4—C5	178.4 (3)
O1^i^—Cu1—N1—C4	178.1 (2)	Cu1—N1—C4—C3	−168.8 (3)
O1—Cu1—N1—C1	−170.8 (3)	C1—N1—C4—C3	1.2 (5)
O6—Cu1—N1—C1	−72.5 (3)	C3—N2—C2—C1	−0.7 (5)
O1^i^—Cu1—N1—C1	9.2 (3)	C2—N2—C3—C4	−0.4 (5)
O6^i^—Cu1—N1—C1	107.6 (3)	Cu2—N2—C3—C4	177.2 (3)
O6^i^—Cu1—N1—C4	−83.6 (2)	Cu2—N2—C2—C1	−178.3 (3)
O3—Cu2—N2—C3	−126.7 (3)	N1—C1—C2—N2	2.1 (5)
O2^ii^—Cu2—N2—C3	−35.5 (3)	N2—C3—C4—N1	0.2 (5)
O2—Cu2—N2—C3	144.5 (3)	N2—C3—C4—C5	−176.6 (3)
O2—Cu2—N2—C2	−38.0 (3)	N1—C4—C5—O1	−14.2 (5)
O3—Cu2—N2—C2	50.8 (3)	C3—C4—C5—N3	−18.3 (6)
O2^ii^—Cu2—N2—C2	142.0 (3)	N1—C4—C5—N3	164.8 (3)
O3^ii^—Cu2—N2—C2	−129.2 (3)	C3—C4—C5—O1	162.7 (4)

Symmetry codes: (i) −*x*, −*y*+1, −*z*+1; (ii) −*x*+1, −*y*, −*z*+1; (iii) *x*, −*y*+1/2, *z*+1/2; (iv) *x*, *y*+1, *z*; (v) −*x*, *y*+1/2, −*z*+3/2; (vi) −*x*+1, −*y*+1, −*z*+1; (vii) −*x*+1, *y*+1/2, −*z*+3/2; (viii) *x*, −*y*+1/2, *z*−1/2; (ix) −*x*+1, *y*−1/2, −*z*+3/2; (x) *x*, −*y*+3/2, *z*+1/2; (xi) *x*, *y*−1, *z*; (xii) −*x*, −*y*, −*z*+1; (xiii) −*x*, *y*−1/2, −*z*+3/2; (xiv) *x*, −*y*+3/2, *z*−1/2.

### Hydrogen-bond geometry (Å, º)

**Table:**

*D*—H···*A*	*D*—H	H···*A*	*D*···*A*	*D*—H···*A*
O2—H2*B*···O4^viii^	0.83 (3)	2.01 (4)	2.845 (4)	177 (5)
O2—H2*C*···O7^vi^	0.83 (4)	2.03 (3)	2.815 (4)	159 (3)
O3—H3*D*···O7^ix^	0.82 (3)	1.87 (3)	2.687 (3)	175 (5)
O3—H3*E*···O4^vi^	0.82 (3)	1.87 (3)	2.679 (4)	169 (3)
N3—H3*B*···O6^xi^	0.86	2.01	2.820 (4)	157
N3—H3*C*···O5^xiii^	0.86	2.03	2.862 (5)	162
C1—H1···O7^xiv^	0.93	2.24	3.108 (5)	154

Symmetry codes: (vi) −*x*+1, −*y*+1, −*z*+1; (viii) *x*, −*y*+1/2, *z*−1/2; (ix) −*x*+1, *y*−1/2, −*z*+3/2; (xi) *x*, *y*−1, *z*; (xiii) −*x*, *y*−1/2, −*z*+3/2; (xiv) *x*, −*y*+3/2, *z*−1/2.
